# Using email interviews to reflect on women’s careers at a regional university

**DOI:** 10.1007/s13384-023-00617-9

**Published:** 2023-03-20

**Authors:** Anitra Goriss-Hunter, Kate White

**Affiliations:** Institute of Education, Arts and Community, Federation University, Mt Helen Campus, PO Box 663, Ballarat, 3353 Australia

**Keywords:** Asynchronous email research, Gender, Higher education, Women’s career progression

## Abstract

The article investigates asynchronous narrative research via email as a flexible and agentic method of collecting data that may empower female participants. A case study was used that focused on the challenges for academic and professional women at an Australian regional university. Twenty-one women responded by email to a range of questions about working conditions and career progression. The data demonstrated that participants found this methodology empowering, encouraging agentic behaviour as they could respond at a time that suited them and in as much detail as they desired. They could also leave their narratives and return to them after some reflection. While lacking the non-verbal markers that often add to meanings in face-to-face interviews, the participants’ writing gave voice and form to their lived experience that has been missing from academic literature. This research method may be vital in the continuing COVID-19 environment where it can be difficult to access geographically dispersed participants.

## Introduction

The authors had been encouraged to undertake this study after analysing Thomas et al.’s (2019) research at another Australian regional university, which explored how working in a regional university with dispersed campuses had an additional impact on women’s career progression. While the participants in the present study were located at a range of dispersed campuses and required flexible interviewing arrangements due to a variety of carer and other family responsibilities, they were also keen to tell their stories. So, as feminist researchers, we were determined to find methods that would enable participants to share their stories of lived experience in their own way and time, without placing any added pressure on their already busy and sometimes complex professional and personal lives. In addition, we needed to access geographically distant participants. We, therefore, adopted the method of asynchronous email interviewing. In this article, we examine its use and explore how it might encourage participants’ agentic and flexible involvement in research projects and also enable the voices of women working at regional universities to be heard among various metro-centric analyses of women in higher education.

The regional university sector in Australia comprises seven universities based in regional cities rather than metropolitan areas. Their peak body is the Regional University Network (RUN, 2021). In recent years regional universities have become even more important in rapidly growing areas outside metropolitan locations that have witnessed the largest net inflow of population over the past two years (ABS, 2021). As there was a gap in the literature on women in regional universities, we considered that this research might begin to address the absence and could also contribute to understanding the critical role of such universities in the growth of regions (Goriss-Hunter & White, 2021; RUN, 2021). For example, regional universities play a vital role in attracting and retaining diverse cohorts—including first-in-family (FiF) and regional and rural students and staff (Goriss-Hunter and Burke (2015).

## Literature review

This literature review explores the range of narrative research that we investigated in our search for a method that would encourage agency, allow flexible options and facilitate the participation of women who worked on dispersed university campuses. As feminist researchers, the authors wanted to use a method that empowered women by enabling them to voice their lived experience, thus making visible those stories that were largely missing in the current literature. In doing so, we aimed to explore an alternative research method that challenged traditional power relations between researcher and participant.

There is a considerable history of feminist criticism of conventional research methods that replicate unequal power relations and reinforce the notion of the researcher as ‘expert’ and authority in the field of knowledge production (Frost & Elichaoff, 2014; Hesse-Biber, 2014; Linabary & Hamel, 2017). This concept of power relations encompasses structural inequities in specific social and cultural contexts as well as within the research process itself: ‘Feminist research then pays close attention to issues of power in the researcher/researched relationship, with goals of interrogating and disrupting unequal power relations as well as recognising participants’ agency and resistance’ (Linabary & Hamel, 2017, p. 99). To enable the ongoing examination of the roles of researcher and participant we combined a feminist lens and a reflexive approach in the asynchronous email method with questions that were open-ended and also focused on particular issues that have been raised in current literature concerning women’s career progression. A reflexive approach to research has been employed by feminist scholars ‘as a key tool for interrogating the impact of the researcher, evaluating the research process and outcomes, and enabling empowerment through the development of a critical consciousness’ (Linabary & Hamel, 2017, p. 99). This ‘continuing mode of self-analysis and political awareness’ (Callaway, 1992, p. 33) emerges through the interrogation of the ways in which issues concerning power might manifest in relationships (Hesse-Biber & Piatelli, 2012). Researchers posit that reflexivity in research projects can be a vehicle for the empowerment of both respondent and researcher (Hesse-Biber, 2014, p. 216). Linabary and Hamel (2017) note that ‘Participants can engage in analytical reflexivity themselves during the research process by evaluating their own experiences and views’ (p. 99). It is through this process in which the participants’ stories of lived experience written in their own words and not contained within the researcher’s written expression, that the conventional power of the interviewer can be challenged (Hesse-Biber, 2014; Linabary & Hamel, 2017). Giving voice to participants renders visible the images of women working in an Australian regional university, previously absent in much of the current literature.

To facilitate this process, we focused on narrative inquiry via email rather than face-to-face interviewing. There are considerable differences between these approaches in terms of the process, product and responses. Face-to-face interviews are in the moment with non-verbal and paralinguistic cues adding to the qualitative data. However, email interviews can be carefully shaped and edited as participants have opportunities to re-read their writing, and revise or delete parts of their narratives.

In the following sections, the literature review investigates various forms of written responses for interviewing purposes including narrative correspondence, email correspondence, asynchronous email interviews and feminist correspondence/writing.

### Narrative correspondence

This method of research gathers and mines written narratives to investigate the lived experience of particular individuals or groups of people (Grinyer, 2004; Milligan, 2005; Milligan & Morbey, 2016). The approach is especially useful in capturing a participant’s own experience using their written narratives. It has been used, particularly in early twenty-first century research, to explore the ideas and feelings of groups that are often difficult to investigate such as the parents of young cancer patients (Grinyer, 2004), mid-life women with long-standing illnesses (Kralik et al., 2001), and older citizens (Milligan, 2005). The method enables participants to write narratives that relate personal stories which are particularly relevant to the individual writer and also speak to the social and cultural context in which they are located. As such, this approach ensures that participants can control the substance and form of the information that is shared.

### Email correspondence

The narrative correspondence method has been expanded as increasingly researchers draw on technology to assist with qualitative methods of data collection including email correspondence as a research approach (Mann & Stewart, 2000; Parris, 2008). It was used by Parris (2008) to build connections between middle managers, who found it ‘a relatively low-cost method of accessing participants in a wide geographical area. Its use allows an opportunity for reflection on research questions via a medium which many employees are now comfortable and competent in using’ (pp. 11–12). So, the method enabled individuals to write their own stories as they unfolded in everyday life while also letting them reflect on their experiences. The challenges involved in using this approach include the considerable time commitment and effort from the researcher to establish rapport with participants. However, Parris (2008) argues that these challenges were balanced by the richness of data collected.

### Asynchronous email interview as a qualitative research method

The asynchronous email interview method is increasingly becoming a popular research approach, particularly for writers who are technologically competent and confident (Bampton & Cowton, 2002; Opdenakker, 2006) as it has a number of benefits (Cook, 2012) such as gathering rich data and encouraging reflexive responses from both participants and researchers (McCoyd & Kerson, 2006). This approach can allow researchers to work with groups that may be difficult to access or where a topic might involve sensitive or complex issues and anonymity may be an important element in the project. In addition, it does not require a large budget.

Although the asynchronous email interview approach has advantages, it can also present some challenges. As researchers are not required to directly interact with participants (Cook, 2012), the absence of nonverbal cues and paralinguistic elements of communication can mean that some nuances of expression are not available (Milligan & Morbey, 2016). The importance of such nonverbal cues is an element that needs to be considered. Also, this method of interviewing does not permit additional follow up questions to be asked at first, so researchers cannot immediately clarify their understanding or pursue avenues of inquiry that occur in the moment.

However, Bjerke (2010) suggests that although the interviewer is not able to see the participant and observe physical and verbal cues that may provide added information, the lack of visibility also means that the researcher then approaches the written words of the respondent without any pre-conceived ideas or unconscious bias. Employing a ‘constructivist-inspired interaction perspective’, Bjerke (2010, p. 1717) suggests that using the asynchronous email interview approach enables the interviewer to draw on ‘other possible strategies of visibility’ that emerge from the interviewing process rather than starting the interview with preconceived ideas about the participant. To do this, researchers must draw on their professional knowledge and personal understanding of the relevant domain to ensure nuanced accounts of participant responses.

### Feminist research – correspondence and email

Historically, using ‘correspondence’ (interviewers communicating by writing letters) has enabled researchers to gather data from geographically distanced participants while establishing a ‘distanced rapport’ (Letherby & Zrodowski, 1995). Even in this earlier literature, the use of letter writing as a methodology was examined as both a general approach and feminist research. Letherby and Zrodowski (1995, p. 576) remarked that ‘at present this method is not often used, even though it provides rich data and is a potential powerful tool for feminist research’.

Qualitative methods of research such as interviewing have been recognised by feminist researchers as useful approaches for examining self-identified women’s experiences in context (DeVault & Gross, 2012). Conventional power structures regarding the researcher and participant can be challenged as both parties can work together to become ‘co-participants’ in generating situated new knowledge (Hesse-Biber, 2014). In an increasingly technological world, technology can be employed to adapt existing qualitative approaches to research to gather rich data electronically and thereby facilitate an empowering method of data collection.

Linabary and Hamel (2017) argue that interviewing by email is potentially an empowering method for feminist researchers and proposed a ‘reflexive email interviewing’ approach that seeks to maintain a feminist lens on research while also addressing the implicit challenges in using an email interviewing method. Based on their own experience, Linabary and Hamel (2017, p. 108) assert that reflexive email interviewing should use the following criteria: (1) the researcher’s use of explicit and implicit strategies to disrupt power hierarchies within the email interview process, (2) the researcher’s engagement in reflexivity of her/his/their own positionality as a continuous part of the research process, and (3) continued invitations for participants to directly reflect on and respond to the research process.

The researchers in the current study found these criteria useful and, therefore, invited the participants to ‘reflect and respond’ by asking them a final question: ‘Do you have any suggestions to reduce these barriers at the University?’ An earlier question had asked ‘What impact has the intersection of gender, personal circumstances, travel, access to professional development/other opportunities and team events had on your career progression’? Some participants interpreted these factors as barriers. So, the responses to the final question often focused on these issues. The impact of using open-ended questions on specific issues that have been identified in the literature as ongoing concerns for women, opened up space for participants to voice their feelings and thoughts about their working lives and careers. They passionately—and often at length—articulated their experiences which impacted on the researchers’ understanding of the issues that have been raised concerning women’s career progression and those concerns that were particular to this study. This approach thus enabled participants to clearly give voice to and make visible the working lives of the women at a regional university.

Consistent with feminist research like Caretta et al., (2018), the authors examined women’s lived experiences of working at a university to create space to explore what issues might impact on career progression and work experiences. As feminist researchers, we understood the potential for this method of asynchronous email research that connected data collection with empowering participants to be particularly useful in a world where restrictions due to COVID-19 continue to have direct and extensive impact on the ways in which we conduct research.

## Research design

After the project was approved by the university’s Human Research Ethics Committee, we collected data from women working at an Australian regional university in February 2020, just weeks before the COVID-19 pandemic impacted on universities across Australia. Women comprise over 70% of professional staff and just over 50% of academic staff (UA, 2020) in the 1000 plus workforce in this university. We define professional staff as those who work in universities but are not academics. Their roles can encompass administrators, but also project managers, librarians, career guidance officers, marketing and professional experience (or placement/work integrated learning) staff.

The principal researcher circulated an email through the university’s daily news service inviting all women staff to participate and provide written responses to a series of questions that examined barriers to and enablers in establishing and progressing their careers. A total of 21 women participated. Eleven of the women were academics and ten were professional staff (often referred to as administrative staff). The response rate was 3.5%.

The low response rate may be explained by a few factors. First, the invitation email was sent out in the daily news email just before we went into lockdown with COVID-19 and the pandemic severely limited women’s capacity to participate in research due to increased professional and domestic workloads. Second, the logistics of managing heavy teaching loads and other work responsibilities at the beginning of semester meant that staff had limited time available for participation in research projects. Nevertheless, the principal researcher chose this time immediately before the first semester as, with their heavy teaching load and carer responsibilities, it was then possible for them to conduct research.

While the number of responses was fairly low, they were enthusiastic in tone and expression. They remind us that there is still a gap in the literature and silence concerning the working lives and career progression of women employed in regional universities. The thoughtful and substantial responses to this project, despite the participants’ considerable workloads, demonstrate that they were keen to tell their stories.

### Research questions

The research questions that elicited these eager responses were divided into two sections. The first was headed identity and asked participants the following: where they grew up, languages spoken at home, if they identified as a carer, and for whom did they provide care? The second section focused on their careers and included questions about factors which they perceived were barriers to, and assisted in, establishing and progressing their careers.

These questions became prompts for self-reflection (Thomas et al, 2019). The written responses were then formed into categories that corresponded with the questions, and data were coded within these main categories, to form sub-categories (Kuckartz, 2019) and analysed. Using this methodology can take longer to analyse the data and ‘derive all the emotional meanings from the text as there is not a face-to-face element’ (Letherby & Zdrodowski, 1995). However, it can be effective for understanding an individual’s experience of events in their own words and where there is little evidence on the topic (Milligan & Morbey, 2016). In addition, the authors argue that the asynchronous email method opens up space for agentic behaviour of women participants.

## Key findings

Ten of the 21 participants grew up in regional Victoria, three grew up in Melbourne, three interstate and four overseas. All spoke English, and five spoke a European language and two spoke a non-European language. Twelve participants identified as carers. Four more said that while they were not currently caring for others, in the past they had been carers. Thus, half of the participants grew up in regional Victoria, all spoke English and over half either identified as currently having caring responsibilities or had been a carer.

We noticed that the participants were mostly not in leadership positions. In addition, they enthusiastically responded to email interviewing, generously writing rich and capacious narratives. Most replied within two to five days of the interview email being circulated through the daily news email service and wrote two to four pages (approximately six hundred to one thousand words) in answer to the questions. Despite heavy workloads and other responsibilities, they had the flexibility to answer in their own time and manner with as much detail as they desired, and they wrote sometimes passionately and at length about issues and circumstances that had impacted on their careers and working conditions, which concurs with Gibson’s (2014) observation. From these full and articulate narratives, we could conclude that the participants were clearly comfortable with email interviews (Parris, 2008). As Milligan and Morbey (2016, p. 106) note, providing written accounts ‘places a greater degree of control over the shape and content of information that the participant chooses’ and the narratives are ‘both personal, in that they are embodied within a specific individual, and social, in that they take their narrative from the context within which they are embedded’. We did not detect any sense that the responses were overworked (Gibson, 2014), possibly because these women generally had multiple paid work and domestic responsibilities and had to carve out time to respond to the questions.

The distinctive voices of participants were certainly apparent, with their narratives enabling a greater visibility of self-identified women working in regional higher education. This increased vocal and perceptible presence was especially evident in relation to roadblocks to their careers and the final question about suggestions for reducing any barriers to women’s careers. The breadth and openness of responses to this final question highlighted how the process facilitated participants’ agency. The open-ended questions that invited participants’ individual thoughts and feelings created space for their voices to be heard and their narratives to be rendered visible.

Participants wrote generally about the roadblocks and barriers to career progression that they experienced and they often gave a few different examples. Their narratives did not focus on wanting to move from one particular role or level of employment to another. However, the data demonstrated that the careers of women with carer responsibilities were negatively impacted by taking time away from work to fulfil those duties as their career ambitions were hindered by a lack of currency when they returned to work.

Four main themes concerning roadblocks and barriers to careers were identified from the data in relation to the final question: unfair working conditions, the need for professional development, travel requirements, and combining work and carer responsibilities. These themes were also reflected in the responses to earlier questions. An analysis of this data revealed that 14 of the 21 participants (66%) discussed theme one—a sense of social justice regarding casualisation and the impact on career development. A higher proportion of participants (95%) discussed theme two about the need for professional development to build capacity and networking opportunities while 81% saw the requirement to travel as onerous and 90.5% discussed the challenges of combining work and carer roles.

### Unfair working conditions

In the narratives, there was a focus on what the participants perceived as the unfairness of working conditions for academic and professional part-time/casual/sessional staff, describing precarious work cultures (Caretta, 2018) and what Richardson et al. (2019) call the ‘double-edged sword’ of sessional teaching. As the research of Richardson et al. (2019) demonstrates, women may choose to work in a sessional capacity during certain times in their careers but this is often superseded by a desire for more security in an ongoing position (Crimmins, 2016). Comments from both academics and professional staff made audible a strong sense of social justice and allowed the reader to construct images of workers who have struggled with issues of entrenched casualization in higher education. Working as a sessional teacher may suit women at certain career stages for a limited period, but Strachan et al. (2012) and Crimmins (2016) found that few wanted to remain as sessional academics.

For example, this academic observed that:

Some sessional[s] have been teaching every semester for years—I do not feel this is reasonable or fair, and devalues their role in the organisation. Every year even 12-month contracts get rejected—even if they are more affordable. This is dangerous for the quality of the staff who will work with us. (Participant 9)

This participant had strong views about how these issues might limit career opportunities. She also clearly identified a link between poor working conditions and the overall quality of the institution’s staff. Through her confident appraisal of her own situation, the broader issues concerning sessional employment in higher education were made visible in a specific and personalised context. One professional staff member argued, in relation to those on contracts, that the university ‘should not have to lose good staff for not offering the security they may need’ (Participant 7).

Working part-time also appeared to be fraught with issues as it is still considered a solution to work-life balance but in reality, a workplace culture that privileges full-time employees can mitigate against career progression for those who choose this option. A professional staff respondent commented that ‘when I first returned on a part-time basis due to my parenting and carer role, my opportunities to take on other roles were limited by my availability. Not many part-time roles are available at the higher HEW [professional staff] levels, nor job-sharing a common practice here’ (Participant 19).

While another professional staff member thought that employment status had not affected her career because she had worked full-time in ongoing positions, she was.

well aware that if I wanted to work at a reduced fraction, it would be viewed negatively and seen as a lack of commitment to the organisation rather than a wish for better work-life balance. Whenever I advocate for my fractional staff or requests for workplace flexibility for my full-time staff (48/52, etc) I am made aware that it’s not the preference of my [manager] and have to fight to maintain fractional staff or implement flexible options (Participant 11).

Continuing this link between rendering visible concerns such as social justice, both academics and professional staff voiced and made visible women’s requirements for flexible working conditions and understanding of these issues in the work place. For example, a professional staff participant emphasised the need for ‘greater tolerance for fractional staff and flexible work conditions. …Educate to not dismiss the work of fractional staff—part-time does not equal uncommitted, unproductive’ (Participant 11) and an academic stated that the university should ‘Give sessional teachers part-time, ongoing contracts, and recognise all the extra work they do at home’ (Participant 5). Articulating and making visible a worker with a strong sense of social justice was further evident in phrases like ‘greater tolerance’ and ‘part time does not equal uncommitted’.

### Need for professional development

Another strong theme demonstrating agency of expression was the need for professional development. The participants argued that this might include: ‘mentoring colleagues in supportive ways and in accountable programmes. Also, opportunities made available to administrative workers to diversify or take on more challenging or interesting work in other areas like research or tutoring’ (Participant 3). And broadening prospects for staff could encompass ‘Communities of practice for exchange of ideas’ being developed and ‘every staff member should be given the opportunity to link in with programmes and projects that they find interesting, particularly outside of their narrow disciplinary sphere’ (Participant 8). The benefit of improving professional development was that such events ‘would help make colleagues closer and create a secure work environment’ (Participant 21). This theme indicated that professional development opportunities would not only enhance participants’ own career prospects, but open up greater collaboration with other staff, as well as providing opportunities to support colleagues by mentoring staff. From these initiatives, the participants suggested that a more collegial and supportive work environment could be achieved.

### Travel requirements

A third theme that emerged from the narratives was the challenges for both academic and professional staff who were often required to travel for work, due to the university’s dispersed campuses, and their non-work responsibilities such as carer roles. For example, one academic commented: ‘I think it is important that this study explores the multi-campus nature of regional universities and the costs involved with travel for training, conferences, etc. Most of those things are held in major cities’ (Participant 6), and another academic noted that this ‘multi campus aspect is very tough for those in carer roles’ (Participant 14). A professional staff member described ‘seminars, conferences, team meetings and so on that I don’t attend because I can’t justify travelling for a couple of hours. I definitely miss out on those, but I feel supported to look for [other] options’ (Participant 7). Participants saw dispersed campuses as a particular challenge for women working at regional universities due to the impact on those who were carers. These challenges in moving between campuses also included the financial cost and time lost for work and for family commitments.

### Combining work with carer responsibilities

The fourth theme identified in the written responses was the challenge of combining work with carer responsibilities. In response to the final question, several participants, both academics and professional staff, suggested initiatives that might enhance working conditions. These included introducing ‘flexitime’ for professional staff ‘allowing varied work hours across a fortnightly or monthly cycle to accommodate family or carer time to be managed in each work unit more effectively, rather than drawing on limited ‘personal leave’’ (Participant 19), improving flexible work which ‘would help increase the work output and motivation’ (Participant 21), and encouraging ‘networking of parenting academic staff to support each other during the child-raising years… [and] Promot[ing] flexible academic work opportunities for parents with young children’ (Participant 15). Some participants had chosen to prioritise family over career and were clear that they needed flexible working conditions. One made the point that male colleagues did not have the same issue with caring responsibilities that women faced in the workplace:

Gender and personal circumstance have been important factors in limiting my career progression in the later years of my working life, As a woman returning to work … there were limited jobs that I could confidently apply for, knowing that I would be called upon at short-notice to leave work sometimes, and not be available outside normal hours as I would have been previously. I had progressed to a dynamic, project-based … position prior to having children, and returned to a part-time admin support role after the parenting break of five years. There has been no career progression since… (Participant 19)

These four themes identified from participant responses to the final question, as well as earlier questions, were all targeted, clearly articulated and offered thoughtful potential solutions to the issues raised. Participants were confident in their responses and knew what could improve their working lives and career progression, and were generous in the answers they provided. They were also thinking about not only their personal working conditions but those of colleagues, especially where they thought such conditions were unfair. Thus, the breadth and thoughtfulness of responses indicated that participants appreciated the opportunity to document their experiences/opinions, as Gibson (2014) has also noted.

## Discussion

When we chose asynchronous email interviews as the methodology for this research study, we were guided by the approach Thomas et al. (2019) had selected to explore issues specific to women working at another Australian regional university. In addition, given the heavy workload of the researchers, it seemed a more efficient means of qualitative data collection.

We were aware of the challenges of this approach. They included an inability to pick up on non-verbal cues (Cook, 2012). Written accounts would not provide the researchers with the silences, hesitancy, excitement, anger, nods of agreement, shrugs and sighs of resignation that can be gleaned from face-to-face interviews. Also, this approach does not usually involve follow up questions for participants. However, there were benefits in not seeing the participant as the lack of visibility meant that we did not have any knowledge of them—what they looked like, how they spoke, their level of confidence or if they appeared energized or overwhelmed by their working life. Rather, we approached their written words without any pre-conceived ideas or unconscious bias (Bjerke, 2010).

In the final question we invited participants to directly reflect on and respond to the research process (Linabary & Hamel, 2017), which we hoped would empower them. We had wondered if this approach would influence the quality of the data generated as we had previously experienced how research participants gave brief written responses that often lacked much depth. However, the results from the current study were encouraging. Most participants provided detailed and thoughtful responses to the final question, as well as to the other questions, and offered a range of views for reducing barriers at the university for women who worked there.

Analysis of the data provided from these asynchronous interviews revealed a richness of views that may not have been gathered if face-to-face interviews had been conducted. The approach taken offered participants questions which were no more or no less than a starting point for thinking about their working lives. How they chose to respond was entirely at their discretion. There was no prompting, no follow up questions, and no requests for clarification of what they had written. In other words, they were in control of the agenda in this project.

What emerged was a certain commonality in their responses especially in relation to the last question. They appeared to be empowered to respond and in a manner that suited them. There were no comments to the effect that the question was not clear or did not make sense, and few participants said that they could not identify with it. In fact, their responses mirrored views they had expressed in answering earlier questions.

Thus, a reflexive email interview research method not only facilitated the collection of rich data for our project, but also enabled agentic behaviour from participants, allowing them to tell their own stories in their own words, in their own time and place. Consequently, their writing gave voice and visibility to the narratives of women working in regional universities that have previously been missing from much of the research on women’s careers in Australian higher education (Goriss-Hunter & White, 2021).

It is clear that COVID-19 may not disappear from our lives in the short-term and that new variants will create continuing challenges in the workplace, including universities, and generate particular challenges for academic researchers. The ongoing uncertainty about lockdowns and face-to-face contact between staff and students during COVID-19, as well as new variants of the infection emerging and possible future variants, means that university staff may temporarily continue to work from home or they may need to self-isolate due to direct or indirect exposure to the virus in the workplace, in the family or in various social settings. Thus, the stresses on university workers have been amplified by the pandemic (Blackmore, 2020), and especially for women academics (Gabster et al., 2020) and professional staff who tend to experience work place precarity (Caretta, 2018) and extended periods of casual, part-time or sessional work despite demonstrating preference for ongoing permanent work (Crimmins, 2016; Richardson et al., 2019; Strachan et al., 2012).

More generally, throughout the pandemic the gendered divide in household work and increased domestic demands have combined to make apparent the extra load that some women may carry. Women have shouldered greater caring responsibilities for children, at times juggling competing responsibilities such as remote schooling for their children, working at home themselves, and caring for the elderly (WGEA, 2020). This may be particularly relevant for women working in regional universities as they have fewer opportunities for seeking jobs outside their current employment and, unlike their metropolitan counterparts, they do not have the option of applying for work at a number of other higher education institutions in the same city.

Therefore, in a continuing and post-COVID world, this method of written response research may be useful in encouraging participant voice and agency (Donald et al., (2020), without the researcher directing the process through face-to-face interviewing.

## Conclusion

The use of an asynchronous email approach to qualitative research enabled participants, women in academic and professional roles, to write their own stories of career pathways in a regional Australian university. From analysis of the data, four main themes were identified. First, a keen sense of social justice was expressed through the writing concerning the ongoing casualisation of the workforce in higher education and the negative ways this had impacted on women’s career progression. Second, the narratives identified a need for professional development to build capacity and mentoring opportunities. Third, the requirement to travel between campuses was challenging and had financial, psychological and physical implications for those who travelled regularly for work. Finally, there were challenges to career progression and work-life balance for women who attempted to combine work and carer roles. These four key issues are specific to the self-identified women participants as their male counterparts generally may not face the same concerns.

The asynchronous email method itself has significant merit. It challenges the traditional social roles of researcher and participant. The 21 women who participated in the study seemed to be empowered by this research methodology. While asynchronous email interviews may not ever fully replace traditional face-to-face interviews (Ratislavová & Ratislav, 2014) the continuing uncertainty created by COVID-19, and the possibility of more lockdowns globally, means that qualitative data collection will now need to explore more flexible approaches. Written accounts can save the expense and time involved in face-to-face interviewing. While the relationship between the researcher and participant is more physically distant in this method (Handy & Ross, 2005), responses are completed privately in the participants’ own time. This can result in detailed, thoughtful, personalized and insightful information that provides rich data and enables the researcher to get to know the participants despite a lack of personal interaction with them. Most importantly, the asynchronous email method enabled women working at a regional university in this case study to give voice and shape to their own lived experiences in an academic terrain where they have been chiefly silent and invisible within the current literature.

## Data Availability

The participants of this study did not give written consent for their data to be shared publicly, so to maintain confidentiality, supporting data is not available.
